# Role of Interleukin-33 in *Staphylococcus epidermidis*-Induced Septicemia

**DOI:** 10.3389/fimmu.2020.534099

**Published:** 2020-10-15

**Authors:** Min Yang, Yiwen Wang, Yonghong Zhang, Yanjun Li, Qifeng Li, Jintong Tan

**Affiliations:** ^1^Department of Neurosurgery, Xinhua Hospital Affiliated to Shanghai Jiao Tong University School of Medicine, Shanghai, China; ^2^Department of Neonatology, Xinhua Hospital Affiliated to Shanghai Jiao Tong University School of Medicine, Shanghai, China; ^3^Department of Pediatric Neurosurgery, Xinhua Hospital Affiliated to Shanghai Jiao Tong University School of Medicine, Shanghai, China

**Keywords:** IL-33, septicemia, *Staphylococcus epidermidis*, prostaglandin E2, interleukin-17A, interleukin-22

## Abstract

Interleukin (IL)-33 is a member of the IL-1 family, which plays an important role in inflammatory response. In this study, we evaluated the effect of IL-33 on septicemia and the underlying mechanisms by establishing a *Staphylococcus epidermidis* (*S. epidermidis*)-induced septicemic mouse model. The expression of IL-33, IL-1α, IL-1β, IL-6, IL-17A, IL-22, and PGE2 were measured by double antibody sandwich enzyme-linked immunosorbent assay, and bacterial colony formation in peripheral blood and kidneys were counted postinfection. The percentages of neutrophils, eosinophils, and inflammatory monocytes were evaluated by flow cytometry, and tissue damage was assessed by hematoxylin and eosin (H&E) staining. The survival of septicemic mice was monitored daily. IL-33 expression was significantly augmented following *S. epidermidis* infection. High IL-33 expression significantly decreased the survival of model mice, and aggravated the damage of lung, liver, and kidney tissues. However, administration of ST2 (receptor for IL-33) to the *S. epidermidis*-infected mice blocked the IL-33 signaling pathway, which elevated PGE2, IL-17A, and IL-22, and promoted healing of organ damage. In addition, ST2 suppressed the mobilization of inflammatory monocytes, but promoted the accumulation of neutrophils and eosinophils in *S. epidermidis*-infected mice. Inhibition of PGE2, IL-17A, and IL-22 facilitated the development of septicemia and organ damage in *S. epidermidis*-infected mice, as well as reducing their survival. Our findings reveal that IL-33 aggravates organ damage in septicemic mice by inhibiting PGE2, IL-17A, and IL-22 production.

## Introduction

Septicemia is a systemic inflammatory response syndrome that occurs when pathogenic bacteria or conditional pathogens invade the blood circulation and multiply in the blood to produce toxins (1). The main clinical manifestations of septicemia are shivering, high fever, tachycardia, tachypnea, rash, articular pain, and hepatosplenomegaly. In addition, prolonged septicemia can lead to multiple organ dysfunction and failure (2). Typical pathogens of neonatal septicemia include *Staphylococcus epidermidis* (*S. epidermidis*), *Staphylococcus aureus, Escherichia coli, Streptococcus pneumoniae*, and *Klebsiella pneumoniae* (3); among which, *S. epidermidis* parasitizes human skin and mucosal surfaces, and has emerged as an important pathogen in patients carrying surgical implants and medical devices (4).

Interleukin (IL)-33 is a member of the IL-1 cytokine family due to structural homology (5). IL-33 binds to the IL-33 receptor complex, which is composed of ST2 and IL-1 receptor accessory proteins, and has a dual role during infection (6). Similar to IL-1β and IL-18, 2 other members of the IL-1 family, IL-33 is firstly synthesized as a 31 kDa precursor, and then cleaved by caspase 1 to generate mature IL-33. However, unlike IL-1β and IL-18, which are mainly implicated in Th1-type immunity, IL-33 has potent immunomodulatory effects in activating Th2-related immune responses, promoting macrophage and neutrophil migration, and inducing degranulation and maturation of eosinophils (7). Notably, IL-33 has been studied in numerous inflammatory disorders, including colitis, rheumatoid arthritis, and allergic asthma, as well as sepsis (8, 9), in which IL-33/ST2 signaling contributes to organ injury and innate immunity (10, 11).

Prostaglandin E2 (PGE2) is a 20-carbon unsaturated fatty acid that is produced by arachidonic acid cyclooxygenase, and represents an important cell growth factor with immuno-regulatory function. In addition to vasodilation, PGE2 relaxes bronchial smooth muscle and has anti-inflammatory effects (12). PGE2 is generally considered to be an effective pro-inflammatory mediator, which is associated with a variety of inflammatory diseases (13). Exogenous PGE2 has also been shown to possess anti-inflammatory properties in rodents and human (14). Previous studies have reported that IL-17A and IL-22 protect against skin infection in mice (15). IL-17A mediates pro-inflammatory effects by activating the host innate immune system (16), and also synergizes with other inflammatory cytokines to augment inflammatory responses. In humans, IL-17A induces the production of the neutrophil-attracting chemokines CXCL8, CXCL1, and CXCL2, as well as other pro-inflammatory cytokines that enhance neutrophil mobilization (17). Unlike most cytokines directed against hematopoietic cells, IL-22 primarily affects non-hematopoietic cells, such as epithelial cells and fibroblasts, in various tissues including the lung, liver, kidney, thymus, pancreas, breast, intestine, skin, and synovium (18). IL-22 has a profound impact on the regeneration of epithelial tissue after injury by inducing epithelial cell proliferation and inhibiting apoptosis (19). Thus, IL-22 mainly serves as a tissue-protective cytokine that facilitates the repair and regeneration of barrier organs, such as the skin, lung, and gastrointestinal tract; however, pro-inflammatory roles of IL-22 have also been reported (20).

In the current study, a septicemic mouse model was established by infecting mice with *S. epidermidis* (ATCC 12228) in order to analyze the effects of IL-33 on the development of septicemia and the production of PGE2, IL-17A, and IL-22.

## Materials and Methods

### BALB/c Mice

A total of 312 BALB/c mice (male, 6–8 weeks, 18–20 g) were purchased from Shanghai SLAC Laboratory Animal Co., Ltd. The mice were housed in an animal room with a 14-h light/10-h dark cycle, temperature of 22 ± 2°C, and humidity of 50%, and were provided with food and water *ad libitum*. All mouse experiments were approved by the Ethics Committee of Xinhua Hospital Affiliated to Shanghai Jiao Tong University School of Medicine, and were performed in accordance with the guidelines of the Xinhua Hospital Affiliated to Shanghai Jiao Tong University School of Medicine.

### *S. epidermidis* Infection Model

The *S. epidermidis* strain ATCC 12228 was amplified and cultured. When a log-phase culture was grown to an optical density of 0.3 at 650 nm, the bacteria were collected, and diluted with an equal volume of phosphate-buffered saline (PBS). The septicemic mouse model was induced by injection of 0.2 ml of bacterial solution (5 × 10^7^ colony-forming units of *S. epidermidis*) into the tail vein of experimental mice.

### Experimental Design

All mice were acclimated at 22 ± 2°C for 1 week prior to the initiation of the experiment. A total of 12 animals in each experiment were randomly chosen as the control group, and the remaining animals were injected with 0.2 ml of bacterial solution into the tail vein as experimental model groups (i.e., septicemic mice). All infected mice were monitored by daily assignment of clinical score, which involved assessments of the progressive worsening of symptoms, including ruffled fur, hunched posture, weakness, and reduced activity (21). Animals that survived to the end of the 7-day observation period, or those that were identified as moribund (defined by pronounced neurologic signs, inactivity, and severe weakness) were euthanized by CO_2_ asphyxiation followed by cervical dislocation.

#### Experiment 1

To evaluate the level of IL-33 in blood specimens, 24 mice were divided into control group and model group (*n* = 12 per group). Blood was collected from both groups 1, 2, 4, and 6 days after infection.

#### Experiment 2

A total of 96 BALB/c mice were randomly divided into four groups: Control group (PBS), model group, IL-33 group (treated with IL-33; Solarbio), and ST2 group (treated with ST2; ACRO) (*n* = 24 per group). Septicemic mice in the IL-33 and ST2 groups were given daily intravenous administration of 1 μg IL-33 and 10 mg/kg ST2, respectively. Six mice in each group were sacrificed at 24 and 48 h in order to determine the effects of IL-33 on IL-17A, IL-22, and PGE2 production in septicemia. The remaining 12 mice in each group were used to investigate the effect of IL-33 on the survival of septicemic mice; all mice were sacrificed on day 7.

#### Experiment 3

A total of 192 mice were divided into control group (PBS), model group, and model group with different stimulations, including IL-33 group (1 μg), ST2 group (10 mg/kg), PGE2 inhibitor group (Pranoprofen, 5 mg/kg; Selleck), anti-IL-17A group (5 μg; Solarbio), anti-IL-22 group (5 μg; Solarbio), and anti-IL-17A + anti-IL-22 group (5 μg of anti-IL-17 and 5 μg of anti-IL-22) (*n* = 24 per group). In addition to the control and model groups, mice in the remaining groups were given daily intravenous administrations. To further investigate the effects of IL-33 on inhibition of IL-17A, IL-22, and PGE2 in septicemia, 6 mice in each group were sacrificed at 24 and 48 h. The remaining 12 mice in each group were used to explore the effect of inhibition of PGE2, IL-17A, or IL-22 on the survival of septicemic mice; all mice were sacrificed on day 7.

### Double Antibody Sandwich Enzyme-Linked Immunosorbent Assay (DAS-ELISA)

The levels of IL-33, IL-1α, IL-1β, IL-6, IL-17A, IL-22, and PGE2 were measured by DAS-ELISA 24 and 48 h after inoculation. Standard solutions or samples (50 μL) were first diluted with carbonate buffer and distributed into the wells of the microtiter plate, followed by incubation for 30 min at 37°C. Following 5 washes with PBS, enzyme labeling reagent (50 μL) was added to each well, and the plates were incubated at 37°C for 30 min. Following incubation, color developer A (50 μL) was added to each well, followed by color developer B (50 μL), and the reaction was left to develop at 37°C for 10 min in the dark. The reaction was stopped after the addition of a stopping solution, and the optical density was read at 450 nm using a microtiter plate reader within 15 min. All ELISA kits were run according to the manufacturer's instructions.

### Histological Processing of Septicemic Mouse Model Tissues

In experiments 2 and 3, 6 mice in each group were sacrificed at 24 and 48 h postinfection. For histologic analysis, the harvested lung, liver, and kidney tissues of sacrificed mice were fixed in 10% formaldehyde and paraffin-embedded. The paraffin tissue sections (4–7 μm) were stained with H&E, and analyzed by light microscopy. Scoring of lung, liver, and kidney histopathology was performed by a pathologist in a blinded manner, as previously described (22). A five-level scoring scale was used: 0 = normal, 1 = minor, 2 = medium, 3 = advanced, and 4 = very severe injury.

Following inoculation, peripheral blood and kidney tissue homogenates were suspended in PBS through nylon mesh. After incubation at 37°C for 48 h, the number of *S. epidermidis* strain was quantified.

### Colony Formation Assay

At 24 and 48 h postinfection, kidney tissues were surgically removed and peripheral blood samples were collected under sterile conditions. Tissues were minced, dissociated, and filtered, and 100 μL of suspension or peripheral blood was placed in the center of an agar plate, which was incubated overnight at 37°C.

### Flow Cytometry

Whole blood was prepared as a peripheral blood mononuclear cell suspension (PBMC). After centrifugation at 1,000 rpm for 5 min, cells (1 × 10^6^) from each group were resuspended in 200 μL of PBS, and then incubated with phycoerythrin (PE)-labeled anti-Ly6G (Invitrogen, 12-9668-82), anti-Ly6C (Invitrogen, 12-5932-82), anti-Siglec-F (Invitrogen, 12-1702-82), fluorescein isothiocyanate (FITC)-labeled anti-CD11b (Invitrogen, 11-0112-85), and DyLight 650-labeled anti-CCR2pAbs (Invitrogen, PA5-23045) for 1 h in the dark at 4°C. After washing twice with PBS, the cells were analyzed on a FACScalibur flow cytometer (BD, Accuri C6), using CELLQUEST software as per manufacturer instructions.

### Quantitative Real-Time Polymerase Chain Reaction (qRT-PCR)

Total RNA was isolated from lung, liver or kidney tissues of mice by TRIzol reagent (Life Technologies, Grand Island, NY, USA), and reversely transcribed into cDNA by following the First Strand cDNA Synthesis Protocols (New England Biolabs, Ipswich, MA, USA). SYBR Green PCR Master Mix (Agilent Technologies, Santa Clara, CA, USA) was used for carrying out qRT-PCR, based on an ABI7300 System (Applied Biosystems, Carlsbad, CA, USA). The following primers were used for qRT-PCR: GAPDH-F: 5′-CTGCCCAGAACATCATCC-3′, GAPDH-R: 5′-CTCAGATGCCTGCTTCAC-3′; PGE2-F: 5′-CTGTCCTTGGTGCGAGTTG-3′, PGE2-R: 5′-GCCAGTGCGATGAGATTCC-3′; IL-17A-F: 5′-CAAACATGAGTCCAGGGAGAG-3′, IL-17A-R: 5′-TGCGCCAAGGGAGTTAAAG-3′; IL-22-F: 5′-CTGCCTGCTTCTCATTGC-3′, IL-22-R: 5′-GCGGTTGACGATGTATGG-3′. The 2^−ΔΔCT^ method was applied for determining the fold changes of target genes at the transcript level.

### Statistical Analysis

Data are represented as mean ± standard error of the mean. All experiments were repeated at least three times. Data were analyzed by GraphPad Prism® Version 7.0 statistical software. Statistical differences between groups were determined by one- or two-way ANOVA. *p*-values <0.05 were considered to indicate statistical significance.

## Results

### IL-33 in the Blood of Septicemic Mice

**Figure 1:**
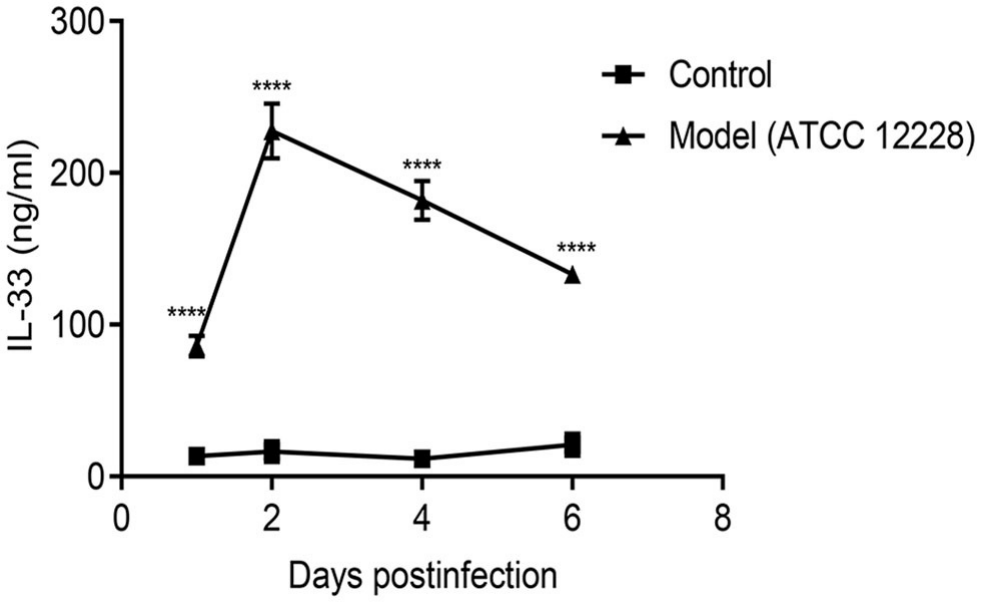
IL-33 level in the blood of septicemic BALB/c mice. A total of 24 BALB/c mice were divided into control group and model group (*n* = 12 per group). The IL-33 level in the blood of septicemic BALB/c model mice was measured by DAS-ELISA (****p < 0.0001, compared to control group).

We first investigated the level of IL-33 in *S. epidermidis*-infected model mice. As shown in Figure 1, IL-33 was significantly increased in the model mice compared to the control group. The greatest increase in IL-33 was observed on day 2, followed by a gradual decrease thereafter.

### IL-33 Inhibits the Production of PGE2, IL-17A, and IL-22

**Figure 2:**
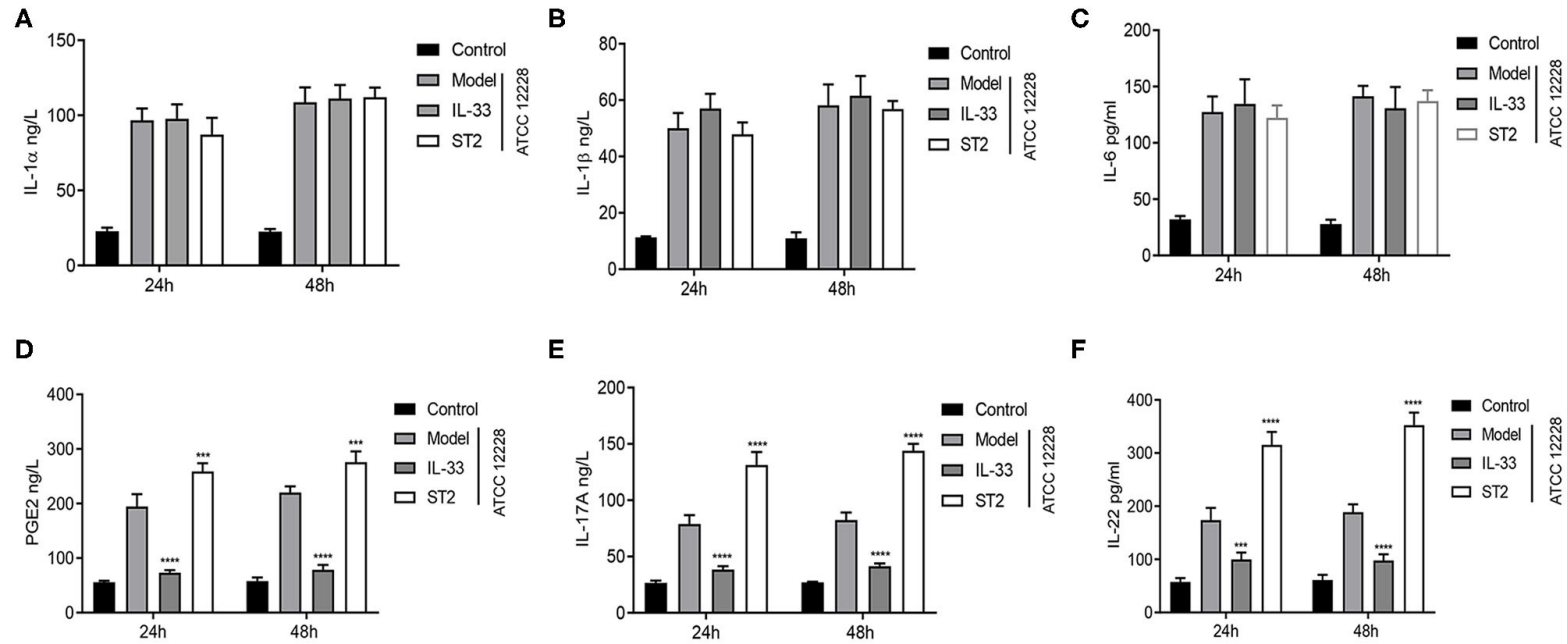
IL-33 inhibits the production of PGE2, IL-17A, and IL-22. A total of 48 BALB/c mice were divided into control group, model group, IL-33 group, and ST2 group (*n* = 12 per group). The levels of **(A)** IL-1α, **(B)** IL-1β, **(C)** IL-6, **(D)** IL-17A, **(E)** IL-22, and **(F)** PGE2 in the peripheral blood of septicemic mice was evaluated via DAS-ELISA at 24 and 48 h postinfection (***p < 0.001, ****p < 0.0001, compared to model group).

Model mice were treated with IL-33 or its receptor ST2 in order to determine the effects of IL-33 on IL-17A, IL-22, and PGE2 in septicemia. There was no statistically significant difference in the levels of IL-1α, IL-1β, and IL-6 among the model, IL-33, and ST2 groups (Figures 2A–C). However, the levels of PGE2, IL-17A, and IL-22 were significantly decreased in the IL-33 group compared to the model group, but increased in the ST2 group (Figures 2D–F). These results suggest that IL-33 inhibits the production of PGE2, IL-17A, and IL-22 in septicemic mice.

### IL-33 Accelerates Bacterial Growth in the Kidney and Peripheral Blood, and Increases Organ Damage in Septicemic Mice

**Figure 3:**
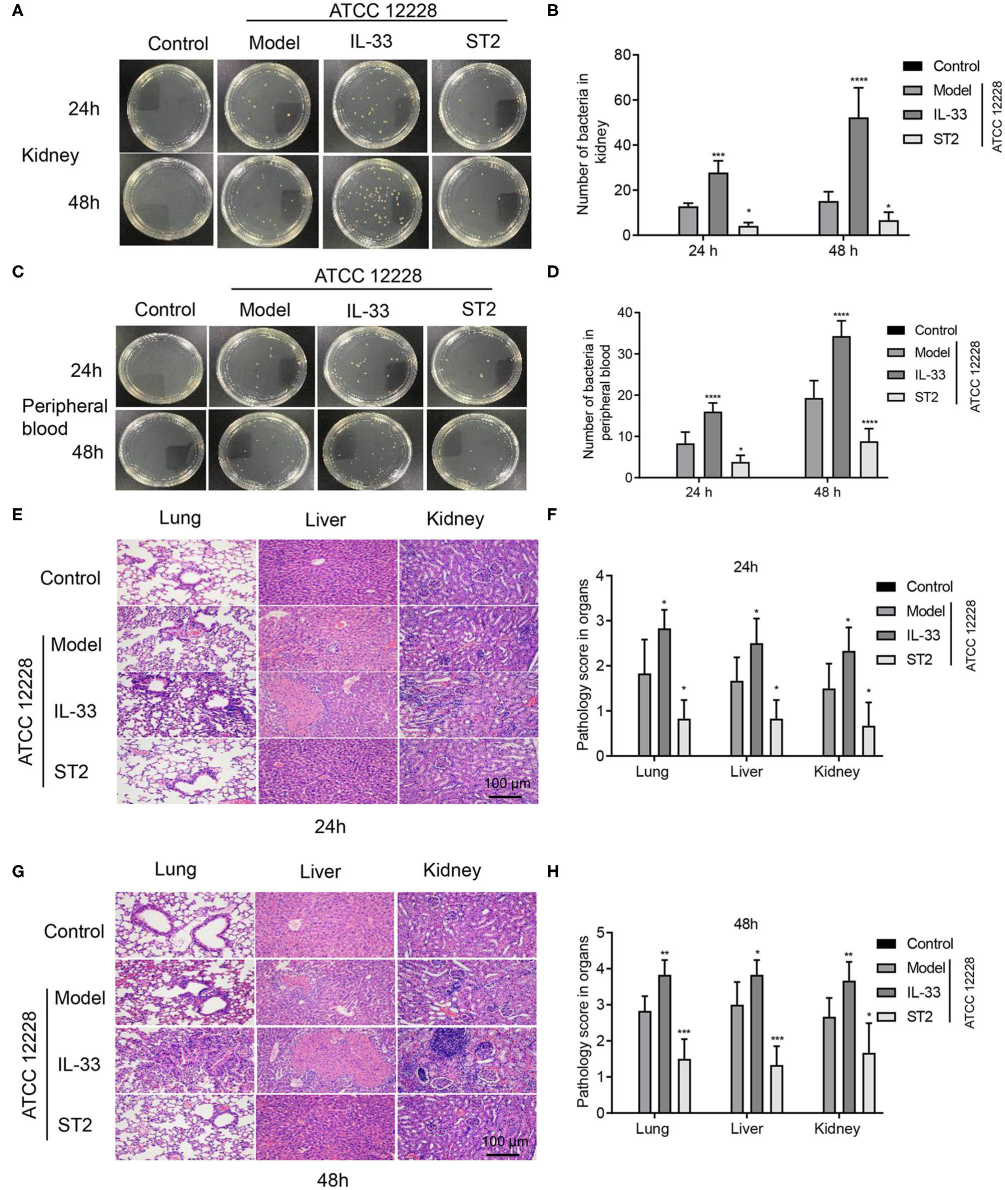
IL-33 accelerates bacterial growth in the kidney and peripheral blood, and exacerbates tissue damage in septicemic mice. A total of 48 BALB/c mice were divided into control group, model group, IL-33 group, and ST2 group (*n* = 12 per group). Colony formation in **(A,B)** kidney tissue homogenate, and **(C,D)** peripheral blood at 24 and 48 h postinfection. H&E staining was used to observe the lung, liver, and kidney injuries at **(E,F)** 24 h and **(G,H)** 48 h (*p < 0.05, **p < 0.01, ***p < 0.001, ****p < 0.0001, compared to model group).

The number of bacteria was assessed in the peripheral blood and kidney of septicemic mice in order to examine the concentration of *S. epidermidis* (ATCC 12228) in each group during infection. The results showed that the number of live bacteria in the peripheral blood and kidney in IL-33-administered mice was significantly higher than that in model group 24 and 48 h post bacterial injection. However, ST2 was shown to suppress bacterial proliferation. Moreover, bacterial reproduction in the kidney homogenate was much faster than that in peripheral blood (Figures 3A–D).

Mice in the model, IL-33, and ST2 groups showed obvious tissue damage 24 h after infection. Compared to the model group, lung, liver, and kidney damage in septicemic mice was more severe in the IL-33 group, but was reduced in the ST2 group (Figures 3E,F). Organ damage in mice was further aggravated 48 h after infection, and IL-33 also increased organ damage at this time point (Figures 3G,H).

### Effect of IL-33 on the Percentage of Inflammatory Monocytes, Neutrophils, and Eosinophils in Septicemic Mice

**Figure 4:**
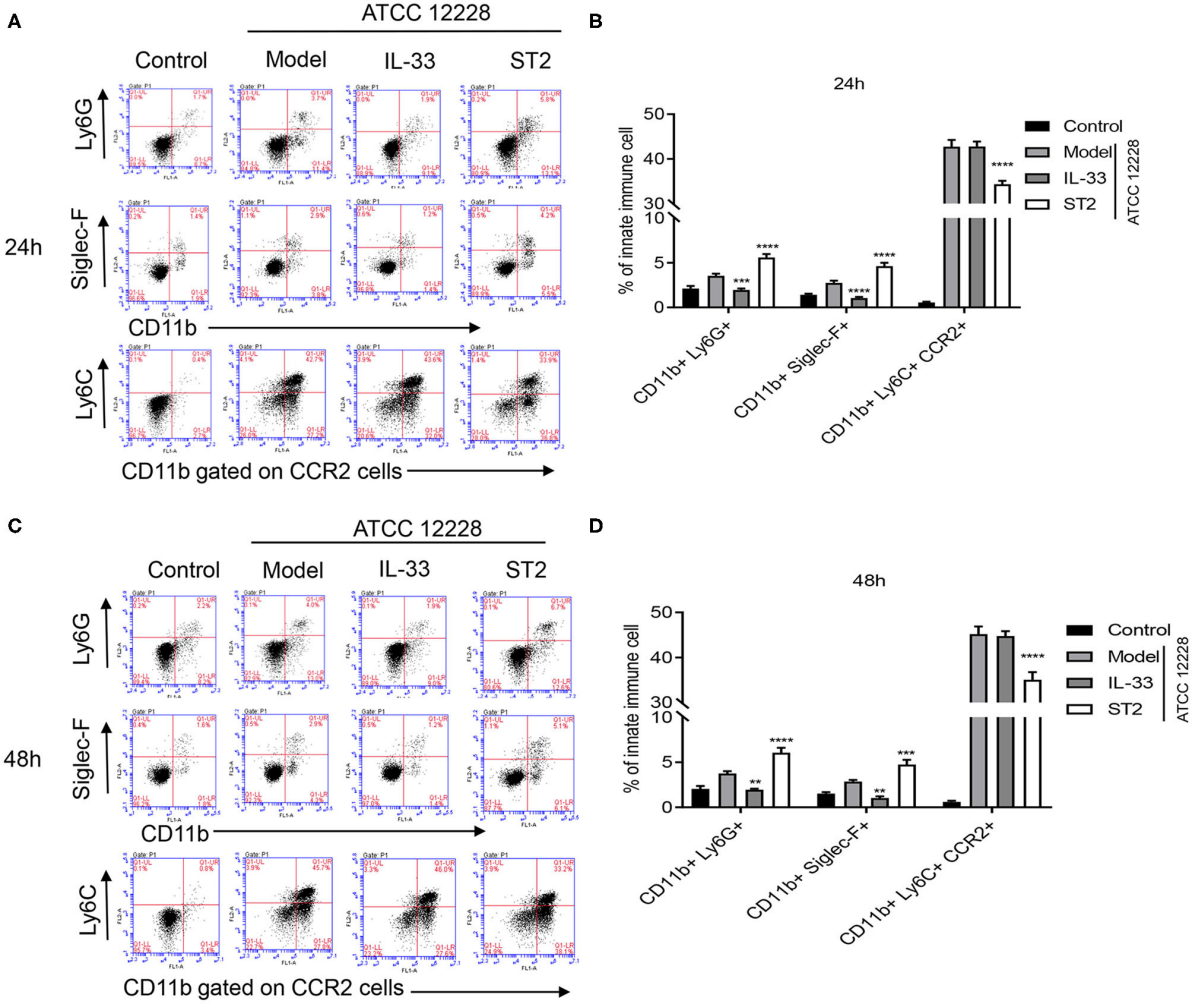
Effect of IL-33 on organ damage and percentage of inflammatory monocytes, neutrophils, and eosinophils in septicemic mice. A total of 48 BALB/c mice were divided into control group, model group, IL-33 group, and ST2 group (*n* = 12 per group). The percentage of peripheral blood neutrophils, eosinophils, and inflammatory monocytes in the peripheral blood of mice was detected at **(A,B)** 24 h and **(C,D)** 48 h postinfection by flow cytometry (**p < 0.01, ***p < 0.001, ****p < 0.0001, compared to model group).

In order to investigate the role of IL-33 on the frequency of innate immune cells, flow cytometry was performed to determine the percentages of neutrophils (CD11b+Ly6G+), eosinophils (CD11b+Siglec-F+), and inflammatory monocytes (CD11b+Ly6C+CCR2+) in the peripheral blood of septicemic mice. The results showed that the percentage of monocytes was much higher than neutrophils and eosinophils postinfection. Nevertheless, ST2 administration significantly reduced the percentage of CD11b+Ly6C+CCR2+ monocytes, but significantly increased the percentage of CD11b+Ly6G+ neutrophils and CD11b+Siglec-F+ eosinophils (Figures 4A–D). These findings suggest that IL-33 inhibition suppressed the mobilization of inflammatory monocytes, but promoted accumulation of neutrophils and eosinophils in septicemic mice.

### IL-33 Inhibition Improves the Survival of Septicemic Mice, as Well as Increases the Expression of PGE2, IL-17A, and IL-22 in Organ Tissues

**Figure 5:**
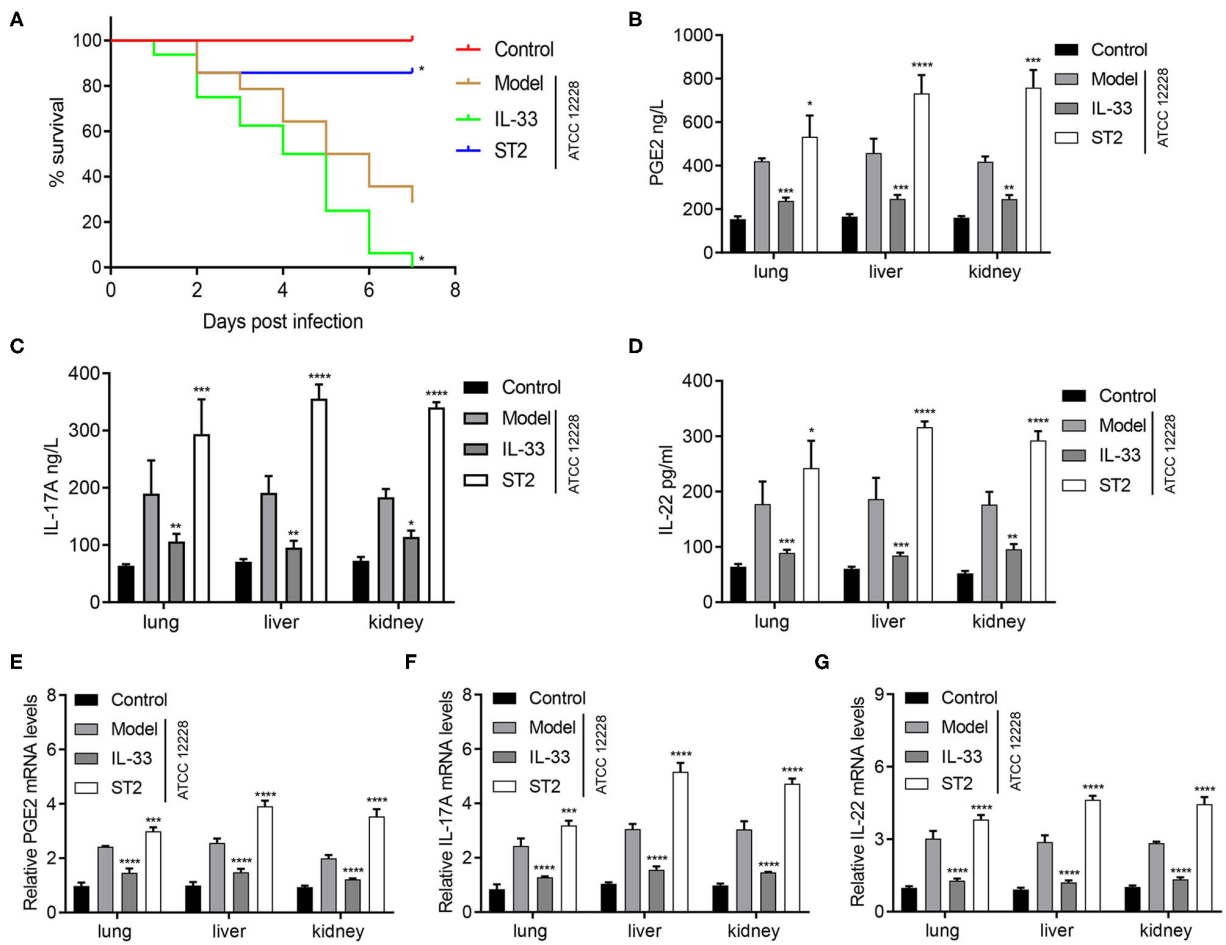
IL-33 inhibition improves the survival of septicemic mice, and increases the expression of PGE2, IL-17A, and IL-22 in organ tissues. A total of 48 BALB/c mice were divided into control group, model group, IL-33 group, and ST2 group (*n* = 12 per group). **(A)** Survival curve of BALB/c mice. Expression level of **(B,E)** PGE2, **(C,F)** IL-17A, and **(D,G)** IL-22 in lung, liver and kidney homogenates were determined via **(B–D)** DAS-ELISA and **(E–G)** qRT-PCR (*p < 0.05, **p < 0.01, ***p < 0.001, ****p < 0.0001, compared to model group).

We next investigated the effect of IL-33 on the survival of septicemic mice. Seven days postinfection, the survival rates were 0, 85.7, and 28.6% in IL-33-treated mice, ST2-treated mice, and model mice, respectively (Figure 5A). In contrast, DAS-ELISA results indicated that in the lung, liver, and kidney of septicemic mice, the expression of PGE2, IL-17A, and IL-22 was dramatically reduced by IL-33 treatment, but was reversed by IL-33 inhibition (Figures 5B–D). Similarly, qRT-PCR results indicated that in the lung, liver, and kidney of septicemic mice, the mRNA levels of PGE2, IL-17A, and IL-22 was dramatically reduced by IL-33 treatment, but was reversed by IL-33 inhibition (Figures 5E–G). Taken together, these results indicate that IL-33 downregulates tissue expression of PGE2, IL-17A, and IL-22 in septicemic mice.

### Inhibition of PGE2, IL-17A, or IL-22 Facilitates Bacterial Growth in Septicemic Mice

**Figure 6:**
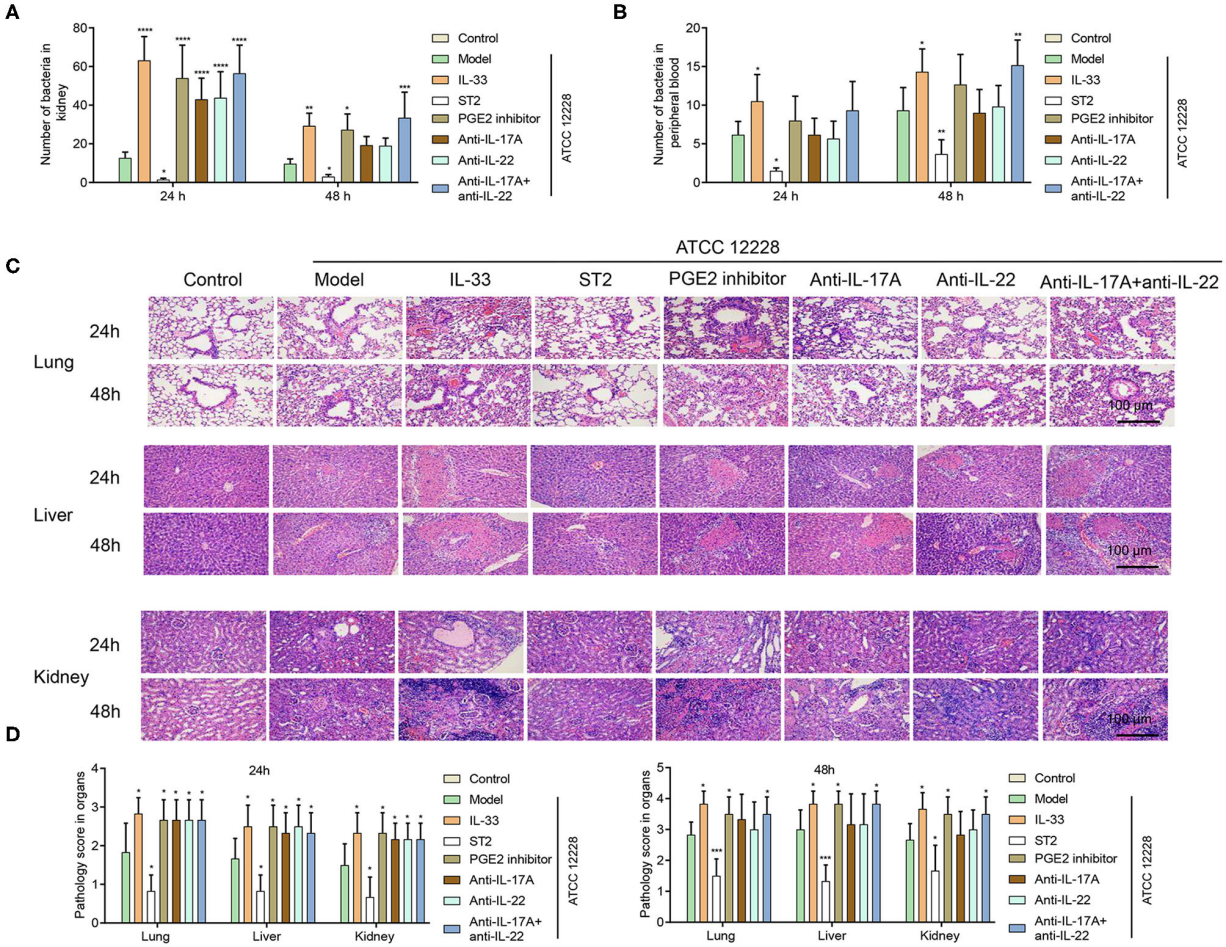
Inhibition of PGE2, IL-17A, or IL-22 facilitates bacterial growth and healing of organ damage in septicemic mice. A total of 96 BALB/c mice were divided into control group, model group, IL-33 group, ST2 group, PGE2 inhibitor group, anti-IL-17A group, anti-IL-22 group, and anti-IL-17A + anti-IL-22 group (*n* = 12 per group). Bacterial numbers in **(A)** kidney tissue homogenate and **(B)** peripheral blood at 24 and 48 h postinfection. **(C,D)** H&E staining was performed to investigate the lung, liver, and kidney injuries (*p < 0.05, **p < 0.01, ***p < 0.001, ****p < 0.0001, compared to model group).

As mentioned above, IL-33 decreased the induction of PGE2, IL-17A, and IL-22, and promoted bacterial growth in the mouse kidney and peripheral blood. Therefore, we speculated that a PGE2 inhibitor, and antibodies against IL-17A and IL-22 might facilitate bacterial growth. As shown in Figure 6A, the number of bacteria in the kidney in IL-33, PGE2 inhibitor, and anti-IL-17A + anti-IL-22 groups was significantly higher than that in the model group at 48 h. Meanwhile, compared to the model group, the number of bacteria in the peripheral blood was only significantly enhanced in the IL-33 and anti-IL-17A + anti-IL-22 groups at 48 h (Figure 6B).

### Effect of Inhibition of PGE2, IL-17A, or IL-22 on Organ Damage and Innate Immune Cells in Septicemic Mice

**Figure 7:**
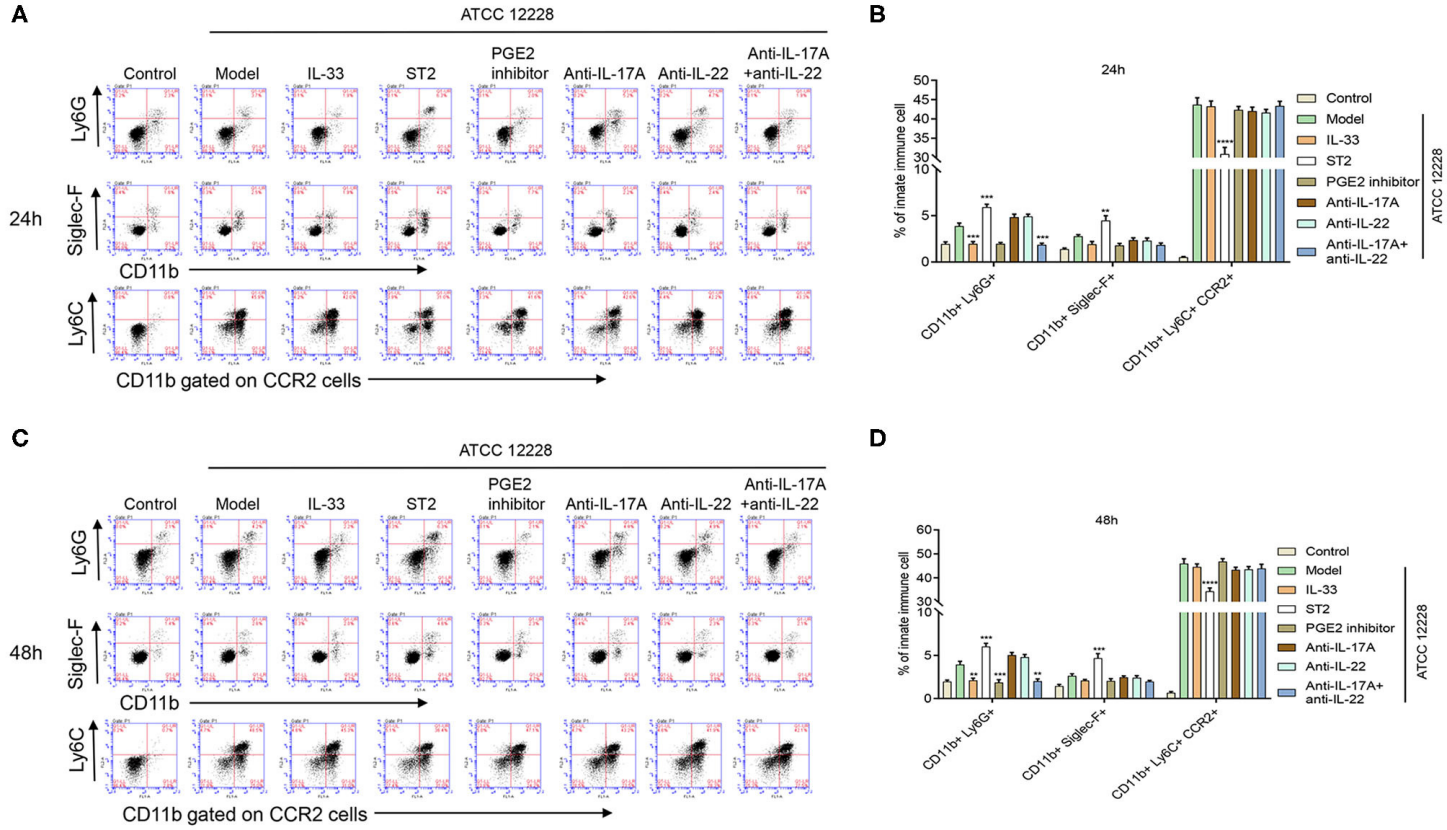
Effect of inhibition of PGE2, IL-17A, or IL-22 on percentages of inflammatory monocytes, neutrophils, and eosinophils in septicemic mice. A total of 96 BALB/c mice were divided into control group, model group, IL-33 group, ST2 group, PGE2 inhibitor group, anti-IL-17A group, anti-IL-22 group, and anti-IL-17A + anti-IL-22 group (*n* = 12 per group). The percentage of peripheral blood neutrophils, eosinophils, and inflammatory monocytes in mice was detected at **(A,B)** 24 h and **(C,D)** 48 h postinfection by flow cytometry (**p < 0.01, ***p < 0.001, ****p < 0.0001, compared to model group).

Compared to the model group, organ damage was alleviated in ST2-treated mice, but was exacerbated in IL-33-treated mice. The inhibition of PGE2, IL-17A, and IL-22 was also shown to promote organ damage in septicemic mice (Figures 6C,D). In contrast, the inhibition of PGE2, IL-17A or IL-22 had no significant impact on the percentage of CD11b+Siglec-F+ eosinophils and CD11b+Ly6C+CCR2+ monocytes at 48 h in the peripheral blood of septicemic mice, while CD11b+Ly6G+ neutrophils were decreased by inhibition of PGE2, IL-17A, and IL-22 (Figures 7A–D).

### Inhibition of PGE2, IL-17A, or IL-22 Reduces the Survival of Septicemic Mice

**Figure 8:**
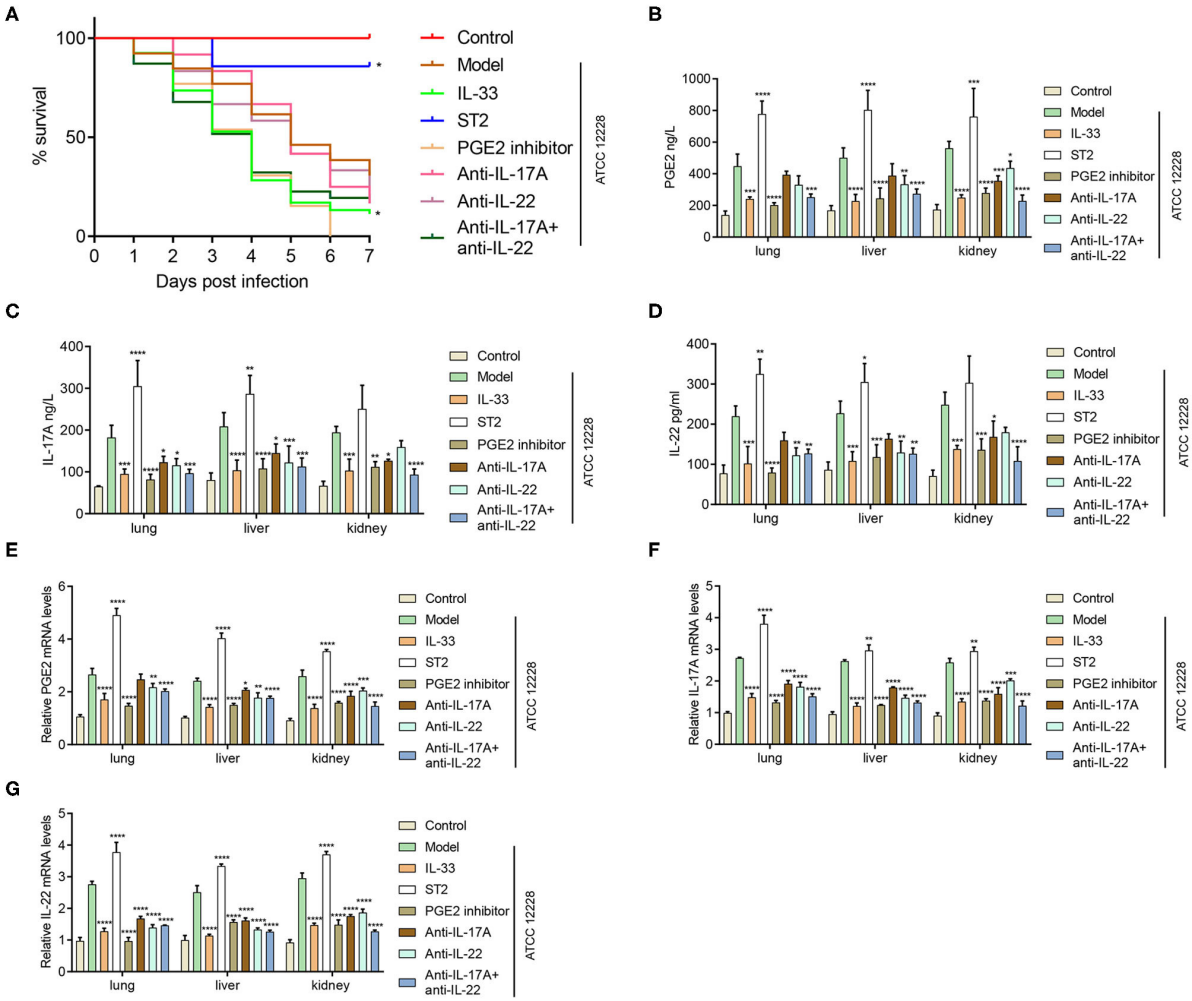
Inhibition of PGE2, IL-17A, or IL-22 reduces the survival of septicemic mice and expression of PGE2, IL-17A, and IL-22 in organ tissues. A total of 96 BALB/c mice were divided into control group, model group, IL-33 group, ST2 group, PGE2 inhibitor group, anti-IL-17A group, anti-IL-22 group, and anti-IL-17A + anti-IL-22 group (*n* = 12 per group). **(A)** Survival curve of BALB/c mice. The expression levels of **(B,E)** PGE2, **(C,F)** IL-17A, and **(D,G)** IL-22 in lung, liver, and kidney homogenates were determined via **(B–D)** DAS-ELISA and **(E–G)** qRT-PCR (*p < 0.05, **p < 0.01, ***p < 0.001, ****p < 0.0001, compared to model group).

As shown in Figure 8A, all septicemic mice treated with the PGE2 inhibitor died 6 days postinfection, and the survival rates in the IL-33, anti-IL-17A + anti-IL-22, anti-IL-17A, and anti-IL-22 groups were 11.3, 19.4, 16.7, and 16.7%, respectively (Figure 8A). In contrast, compared to the model group, the expression of PGE2, IL-17A, and IL-22 in lung, liver, and kidney homogenates were remarkably reduced by IL-33 administration, PGE2 inhibition, IL-17A inhibition, and IL-22 inhibition, measured by DAS-ELISA (Figures 8B–D) and qRT-PCR (Figures 8E–G).

## Discussion

In the current study, we found that IL-33 expression was elevated after *S. epidermidis* infection. In addition, high levels of IL-33 significantly aggravated damage to lung, liver, and kidney tissues in mice. In contrast, administration of ST2 to the septicemic mice blocked the IL-33 signaling pathway, and consequently elevated PGE2, IL-17A, and IL-22, which promoted healing of organ damage. Although PGE2, IL-17A, and IL-22 were decreased following injection of IL-33, they were not completely eliminated. Moreover, IL-33 treatment did not significantly affect IL-1α, IL-1β, and IL-6 production. As a result, we emphasized the function of PGE2, IL-17A, and IL-22 due to their ability to protect and regenerate epithelial tissues (19, 23, 24).

Based on the above results, we conducted a more in-depth study and divided animals into control, model, IL-33, ST2, PGE2 inhibitor, anti-IL-17A, anti-IL-22, and anti-IL-17A + anti-IL-22 groups. Previous studies reported that ST2 treatment reduced inflammation and lethality in hepatic and intestinal ischemia/reperfusion injury (25, 26), and attenuated the inflammatory responses in allergic lung inflammation (27, 28). Similarly, in the present study, flow cytometry was used to detect the percentage of peripheral blood neutrophils, eosinophils, and inflammatory monocytes in septicemic mice. Our results demonstrated that the number of inflammatory monocytes decreased the most following ST2 treatment. Furthermore, it was found that administration of IL-33, PGE2 inhibitor, anti-IL-17A, anti-IL-22, and anti-IL-17A + anti-IL-22 significantly increased tissue damage as a result of the decrease in PGE2, IL-17A, and IL-22. Chan. et al. (15) established a subcutaneous skin/skin structure abscess model that utilized a Methicillin-resistant *S. aureus* (MRSA) strain, and found that IL-17A and IL-22 helped to control the severity of abscesses; nevertheless, inhibition of IL-17A or IL-22 alone resulted in larger lesion areas (skin necrotic areas). Consistent with the results of this study, after 7 days of infection, the anti-IL-17A, anti-IL-22, and anti-IL-17A + anti-IL-22 groups showed more severe organ damage than the model group. However, higher levels of IL-17A and IL-22 alleviated organ damage. Moreover, IL-33 production was increased in septicemic mice compared to control mice, and IL-33 was found to increase the 7-day mortality of *S, epidermidis*-infected mice. This may be due to the fact that increased IL-33 might exacerbate organ injuries caused by inflammation after infection. The current study demonstrated that IL-33 was increased in septicemic mice and exerted great predictive value for septicemia risk in the early phase. However, previous studies have shown that non-detectable levels of IL-33 and soluble ST2 levels above the median dramatically increased the 30-day mortality risk in critically ill patients (29), and that serum ST2 levels correlate with sepsis severity (30). These data suggest dual predictive value of IL-33 and ST2 in patients, dependent on the phase and severity of infection. Therefore, further investigations are warranted to elucidate the predictive value and function of IL-33 and ST2 during the immune response in both mice and patients.

An interesting finding in the current study was that IL-33 negatively regulated PGE2, IL-17A, and IL-22. To date, relatively few cytokines have been identified as negative regulators of IL-17A and IL-22. Among them, TGF-β, the most recognized cytokine, is involved in promoting IRF4-mediated inhibition of IL-17A and IL-22 (19). Others include IL-27, which promotes c-Maf-mediated inhibition of IL-17A and IL-22 (31), and IL-25, which supports the induction of type 2 cytokines that suppresses IL-17A and IL-22 (32). A study by Garth et al. showed that the absence of IL-1RL1/ST2 signaling leads to increased production of IL-17A and IL-22, while elevated IL-33 decreased production of IL-17A and IL-22; these findings indicate that IL-33 signaling negatively regulates IL-17A and IL-22 by IL-1RL1 (33), which was similar to the results of our findings.

Compared to the anti-IL-22 and anti-IL-17A groups, septicemic mice with intravenous injection of PGE2 inhibitor had more severe organ damage after 48 h, which may indicate that PGE2 plays a greater role in protecting organ damage. The role of PGE2 in the production of IL-17A and IL-22 has not yet been elucidated. However, it has been reported that PGE2 may promote the production of IL-17A by previously polarized Th17 cells or memory Th17 cells (34, 35), although the latest data indicate that PGE2 has the opposite effect on Th17 produced by naive T cells (36). Several studies have demonstrated that anti-CD3 activation of human PBMCs promotes IL-17A production in the presence of exogenous PGE2, but significantly inhibits IL-22 production (37). However, a recent report showed that the COX2/PGE2 axis promotes the production of congenital IL-22 (38). In the current *S. epidermidis* mouse model, PGE2 inhibitor was found to cause a decrease in IL-17A and IL-22, suggesting that IL-17A and IL-22 were also reduced in the presence of PGE2. Based on the pro-proliferation, anti-apoptosis, anti-infection, and anti-inflammatory effects of PGE2, IL-17A, and IL-22 (12, 15, 17, 19), these cytokines primarily function as tissue-protective cytokines, which facilitate the repair and regeneration of barrier organs, and inhibit septicemia-induced morbidity and mortality. However, further studies are warranted to investigate the underlying mechanisms of IL-33, PGE2, IL-17A, and IL-22 in septicemia. Furthermore, we found that the severity of organ damage increased as the number of bacteria increased. In addition, the bacterial reproduction in the kidney tissue homogenate was much faster than that in the peripheral blood.

## Conclusion

In the current study, we demonstrate for the first time that IL-33 plays a role in regulating the production of PGE2, IL-17A, and IL-22 in septicemia. In line with this, inhibition of the IL-33 signaling pathway resulted in elevated expression of PGE2, IL-17A, and IL-22, which led to accelerated healing of organ damage. Based on the results of the three experimental observations, we prove that IL-33 aggravates organ damage in septicemic mice by inhibiting PGE2, IL-17A, and IL-22 production.

## Data Availability Statement

All datasets presented in this study are included in the article/supplementary material.

## Ethics Statement

The animal study was reviewed and approved by Xinhua Hospital Affiliated to Shanghai Jiao Tong University School of Medicine.

## Author Contributions

MY and YW designed the study. YW and YZ performed the experiments. YL and QL collected, analyzed, and interpreted the data. MY and JT prepared the manuscript. All authors contributed to the article and approved the submitted version.

## Conflict of Interest

The authors declare that the research was conducted in the absence of any commercial or financial relationships that could be construed as a potential conflict of interest.
